# Influenza Vaccination Coverage among Adults in Korea: 2008–2009 to 2011–2012 Seasons

**DOI:** 10.3390/ijerph111212162

**Published:** 2014-11-25

**Authors:** Hye Jung Yang, Sung-il Cho

**Affiliations:** 1 Graduate School of Public Health, Seoul National University, 1 Gwanak-Ro, Gwanak-Gu, Seoul 151-742, Korea; E-Mail: inmytwenties@snu.ac.kr; 2 Graduate School of Public Health and Institute of Health and Environment, Seoul National University, 1 Gwanak-ro, Gwanak-gu, Seoul 151-742, Korea

**Keywords:** influenza, vaccination coverage, adults, Korea

## Abstract

The aim of this study was to examine seasonal and pandemic influenza vaccination coverage in adults from the 2008–2009 season to the 2011–2012 season, including pandemic and post-pandemic seasons in Korea. We collected data of self-reported vaccine use from the Korean Community Health Survey. We also collected information on socioeconomic status and health behaviors in subpopulations. We tested for linear trends among the data to investigate vaccine coverage before and after the pandemic; and multiple logistic regression analyses were performed to identify predictors of obtaining the influenza vaccination. The results revealed a steady increase in vaccination coverage in every subgroup during four consecutive seasons. The highest rate of vaccine coverage (43.6%) occurred two years after the pandemic. Factors associated with vaccine receipt were: older age; lower education level; lower income; and health behaviors such as regular walking and receiving a health check-up. Smoking and drinking alcohol were inversely associated with vaccination. Having a chronic health condition was also a strong predictor of vaccine receipt. Though vaccination coverage rates were high in high-risk groups; disparities in coverage rates were substantial; particularly in young adults. Interventions are needed to minimize the coverage gaps among subgroups and to improve overall vaccination rates.

## 1. Introduction

Seasonal influenza affects 5% to 10% the global population each year [1] and influenza epidemics cause substantial hospitalizations and complications [2,3]. Young children, the elderly, pregnant women, and people of any age with comorbid conditions such as diabetes, cardiovascular diseases, human immunodeficiency virus, lung disease, kidney disease, and asthma, are more vulnerable to infection and are, therefore, defined as high-risk groups [4,5,6,7,8]. The primary strategy for protection against influenza and prevention of complications associated with the infection is the influenza vaccine [5]. Age and immunocompetence of individuals affect the efficacy and effectiveness of the vaccine, but the World Health Organization (WHO) recommends annual vaccination for all age groups [9,10,11,12,13,14]. Vaccination in high-risk groups, in particular, reduces morbidity and mortality [15,16,17,18,19].

Korea implemented a National Immunization Program (NIP) to increase vaccination against epidemic and pandemic influenza and provide free vaccination to priority groups, which are listed in Table 1 [20]. As part of the NIP, seasonal influenza vaccines are provided each year during the flu season. During the 2009–2010 season, both pandemic and seasonal vaccines were distributed and individuals chose to receive either one or both of the vaccines. Consequently, coverage rates for people aged 65 and older were markedly higher than the average coverage rates in other countries reported by the Organization for Economic Cooperation and Development [21]. During the same season, rates of vaccination for the A(H1N1)pdm09 strain of influenza remained low in young adults in Korea [22].

**Table 1:** Priority groups for seasonal and pandemic influenza vaccination.

Seasonal	Pandemic (2009–2010 Season only)
1. Individuals over 65 years old 2. Financially vulnerable persons 3. Handicapped individuals 4. Soldiers	1. Healthcare workers
	2. Infants and pregnant women
	3. Individuals over 65 years old
	4. Individuals in high-risk groups aged 50 to 64 years
	5. Students (elementary, middle, and high school)
	6. Soldiers
	7. Teachers (preschool and K-12)
	8. Airport, infrastructure, and port workers
	9. Social welfare and child care center residents and workers

A few studies have reported characteristics associated with vaccine receipt in limited populations within Korea [23,24,25], but no studies have addressed vaccination coverage in all adult age groups before and after the influenza pandemic in Korea. The objectives of this study were to describe the trends in seasonal and pandemic influenza vaccination in adults from the 2008–2009 season to the 2011–2012 season and to define demographic factors associated with vaccine receipt.

## 2. Methods

### 2.1. Data Source

For this study, we collected data from the Korean Community Health Survey (KCHS), which was conducted by the Korea Centers for Disease Control and Prevention (KCDC). KCDC has been conducting the annual, nationwide, cross-sectional survey since 2008 and, on the basis of this information, has created health indices of individuals that are comparable among different regions in the country. The sample size for the KCHS is 900 subjects in each of 253 community units, including 16 metropolitan cities and provinces. The KCHS expects a total of 227,700 survey participants each year, but the actual number of respondents is nearly 230,000 [26]. The KCHS uses a two-stage sampling process. The first stage selects a sample area (tong/ban/ri) as a primary sample unit, which is selected according to the number of households in the area using a probability proportional to the sampling method. In the second stage, the number of households in the selected sample tong/ban/ri is identified to create a household directory. Sample households are selected using systematic sampling methods. This process is used to ensure that the sample units are representative of the entire population [27]. For the sample to be statistically representative of the population, the data collected from the survey is weighted based on the sample design [28]. Non-responses and individuals who provide insufficient information on socio-demographic variables and influenza vaccination are excluded and not replaced [27].

The survey is offered only to individuals who provide written informed consent, so their responses might cause volunteer bias to the survey and to the data collected, which would result in positive outcomes for health-related variables. KCHS uses interviewers trained in computer-assisted personal interviewing techniques to collect information. The aim of the survey is to collect information on health behaviors, chronic diseases, vaccination, use of health services, quality of life, and socio-demographic characteristics including gender, age, income, education level, occupation, residency area, and family size. For our study, we used KCHS data from the 2008–2009 season to the 2011–2012 season. 

### 2.2. Study Variables

Socio-demographic variables that we examined in our study included sex (male, female), age group (19–44, 45–64, 65–74, ≥75 years), monthly income (≥2 million Korean won, <2 million Korean Won; 1000 Korean Won = approximately 1 USD), occupation (professional, service/manual worker, others), education level (at least high school, less than high school), residency (metropolitan, urban, rural), and number of chronic illnesses (none, one, two or more). Chronic illnesses included diabetes, asthma, angina, myocardial infarction, stroke, and tuberculosis; all of these are comorbidities for which the KCDC recommends influenza vaccination. The chronic illness variable was defined as a person who had one or more high-risk medical conditions and was receiving medication for treatment at the time of survey completion. Questions included “Have you ever been told by a doctor that you had chronic disease?” and “Are you currently taking any medication for that disease?” Health behavior variables included receiving a health check-up, smoking, drinking alcohol, and regular walking. A health check-up was measured by a single answer (yes/no); this variable was excluded from the survey during the 2010–2011 season. Smoking was divided into three categories: current smoker, former smoker, and never smoker. Regular alcohol consumption was defined as drinking alcohol more than once a month. Regular walking was defined as walking more than 30 min a day at least 5 days a week. Flu seasons last from December to April in Korea and influenza vaccination is recommended from October to December [20]. Therefore, receipt of the influenza vaccination was assessed by asking if the survey participant received a seasonal influenza vaccination (yes/no) in the previous 12 months, which corresponded to September through August and included the winter and early spring peak seasons of epidemics. In the 2009–2010 season, seasonal and pandemic vaccinations were available; in the 2008–2009, 2010–2011, and 2011–2012 seasons, only seasonal influenza vaccination was available.

### 2.3. Statistical Analysis

We performed the χ^2^ test to compare vaccination coverage rates among the seasons. We measured annual changes in influenza vaccination coverage using the χ^2^ test for linear trends. A *p*-value <0.05 was considered statistically significant. We performed multivariable logistic regression to calculate the adjusted odds ratios; we included age, sex, and other variables that showed a *p*-value <0.05 in bivariate analysis. Results are expressed with a 95% confidence interval (CI).

All statistical analyses were performed with SAS statistical software (version 9.3, SAS Institute, Cary, NC, USA). The SAS survey procedure was used to account for the complex sampling design of the KCHS. All analyses were weighted to reflect the age and sex of the Korean population.

## 3. Results

### 3.1. Influenza Vaccination Coverage in Four Consecutive Seasons

**Figure 1:**
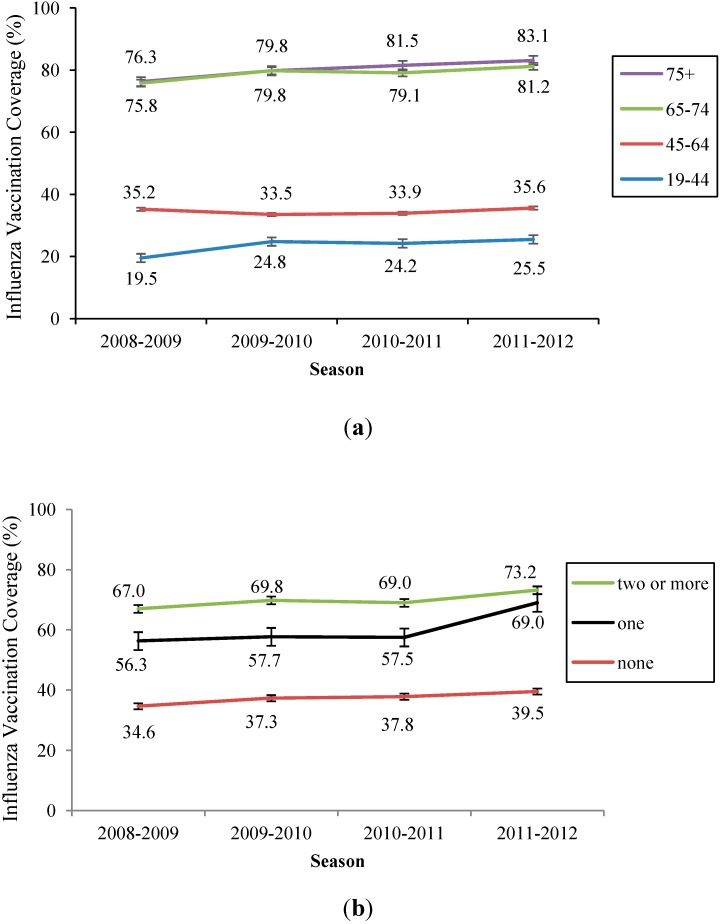
(**a**) Influenza vaccination coverage by age group; (**b**) Influenza vaccination coverage according to number of chronic illnesses.

Influenza vaccination coverage (IVC) rates increased during the study period, with overall rates of 37.8% in 2008–2009, 41.0% in 2009–2010, 41.6% in 2010–2011, and 43.6% in 2011–2012 (Table 2). The highest increase in IVC rates occurred between the 2008–2009 and 2009–2010 seasons. IVC rates were higher in vulnerable groups including people with lower income, people with physical jobs, and people with a lower education level. Notably, individuals with lower income showed the highest increase in IVC rates during the four seasons included in our study, increasing steadily from 46.5% in 2008–2009 to 56.1% in 2011–2012. Vaccination rates increased with age and exceeded 80% in the elderly after the pandemic (Figure 1a). IVC among adults with chronic diseases also increased (Figure 1b). Health behaviors such as not smoking, not drinking alcohol, regular walking, and receiving a health check-up were associated with higher IVC rates. Differences in vaccination rates among subgroups were statistically significant (*p* < 0.05) for each variable.

### 3.2. Determinants of Influenza Vaccination Receipt

For seasonal and pandemic IVC, the following factors predicted a higher likelihood of vaccine receipt according to multiple logistic regression analysis (Table 3): female gender, elderly age group (65–74 years old and ≥75 years old), low income, engaging in service/physical work or other jobs, receiving a health check-up, and having a chronic illness. In the 2011–2012 season, participants in the ≥75 years old age group were the most likely to receive a vaccine (OR 8.12, 95% CI 7.75–8.50), followed by the 65–74 years old age group (OR 6.23, 95% CI 6.00–6.48). Receiving a health check-up was also a strong predictor for IVC (OR 2.28, 95% CI 2.23–2.33). Those who self-reported as a current smoker or regular alcohol drinker were less likely to receive a vaccination than never and former smokers and non-drinkers, respectively.

**Table 2:** Demographic characteristics and influenza vaccination rates from the 2008–2009 season to the 2011–2012 season.

Variables	2008–2009 Seasonal	2009–2010 Seasonal and Pandemic	2010–2011 Seasonal	2011–2012 Seasonal	Test for linear trend *
	Number of Individuals (vaccination Coverage, %)				*p*-value
**Socioeconomic variables**					
Sex					
Male	107,080 (32.4)	104,575 (36.6)	103,017 (36.5)	102,898 (38.4)	<0.001
Female	123,635 (42.6)	124,654 (44.7)	126,209 (45.8)	126,023 (47.8)	<0.001
Age (years)					
19–44	95,611 (19.5)	90,970 (24.8)	87,472 (24.2)	85,060 (25.5)	<0.001
45–64	83,419 (35.2)	84,005 (33.5)	85,130 (33.9)	85,757 (35.6)	<0.001
65–74	33,958 (75.8)	34,823 (79.8)	35,050 (79.1)	35,483 (81.2)	<0.001
≥75	17,727 (76.3)	19,431 (79.8)	21,574 (81.5)	22,621 (83.1)	<0.001
Income ^†^					
≥2000 per month	109,699 (29.2)	104,669 (35.5)	109,798 (31.7)	119,165 (33.6)	<0.001
<2000 per month	112,716 (46.5)	103,916 (49.8)	101,784 (52.3)	96,476 (56.1)	<0.001
Occupation					
Professional	72,118 (24.7)	68,231 (28.0)	70,694 (27.5)	71,868 (29.3)	<0.001
Service/physical	66,141 (39.7)	60,208 (41.5)	70,925 (42.9)	73,042 (45.5)	<0.001
Others ^‡^	92,294 (46.8)	86,883 (50.3)	87,267 (52.0)	83,800 (54.2)	<0.001
Education level					
At least high school	123,800 (25.9)	124,649 (29.3)	124,178 (29.3)	125,419 (31.2)	<0.001
Less than high school	106,646 (51.7)	103,956 (55.1)	104,557 (56.2)	103,069 (58.7)	<0.001
Residency					
Metropolitan	68,647 (30.7)	67,747 (34.6)	67,921 (35.6)	67,852 (37.4)	<0.001
Urban	61,499 (31.5)	60,947 (34.5)	60,837 (35.4)	61,903 (36.9)	<0.001
Rural	97,311 (47.1)	97,318 (49.6)	97,234 (49.9)	94,991 (52.5)	<0.001
Chronic illness ^§^					
None	198,682 (34.6)	191,414 (37.3)	189,346 (37.8)	188,180 (39.5)	<0.001
One	27,566 (56.3)	31,777 (57.7)	33,473 (57.5)	34,120 (60.2)	<0.001
Two or more	4467 (67.0)	6,038 (69.8)	6407 (69.0)	6621 (73.2)	<0.001
**Health behaviors**					
Health check-up ^††^					
No	88,572 (24.3)	86,883 (29.8)	-	76,567 (30.7)	<0.001
Yes	142,143 (46.3)	142,346 (47.9)	-	152,354 (50.1)	<0.001
Smoking					
Never smoker	144,588 (40.7)	144,859 (43.6)	143,886 (44.3)	144,249 (46.2)	<0.001
Former smoker	31,168 (44.5)	33,551 (47.8)	36,612 (47.9)	36,831 (50.9)	<0.001
Current smoker	54,779 (26.3)	50,741 (29.2)	48,679 (28.9)	47,812 (29.9)	<0.001
Drink alcohol					
No	114,699 (46.6)	113,314 (49.6)	111,358 (50.8)	112,986 (53.0)	<0.001
Yes	115,931 (29.1)	115,726 (32.6)	117,743 (32.9)	115,880 (34.4)	<0.001
Regular walking					
<30 min/5days/week	113,656 (36.6)	129,781 (40.2)	133,215 (40.8)	136,324 (43.2)	<0.001
≥30 min/5days/week	116,896 (39.0)	98,988 (42.1)	95,576 (42.7)	92,343 (44.2)	<0.001
**Total**	**230,715 (37.8)**	**229,229 (41.0)**	**229,226 (41.6)**	**229,921 (43.6)**	**<0.001**

Notes: * χ^2^ for linear trend between the 2008–2009 and 2011–2012 seasons; ^†^ Units: Korean Chun-Won (₩1000); ^‡^ Others include soldiers, college students, and housewives; ^§^ Patients were diagnosed with one or more of the following diseases by a doctor and were receiving a medication for the disease: diabetes, stroke, heart disease (angina or myocardial infarction), hepatitis, or tuberculosis; ^††^ Variable was excluded from the questionnaire in the 2010–2011 season.

**Table 3:** Adjusted odds ratios of demographic characteristics associated with influenza vaccine receipt from the 2008–2009 season to the 2011–2012 season.

Variables	2008–2009		2009–2010		2010–2011		2011–2012	
	Adjusted OR	95% CI	Adjusted OR	95% CI	Adjusted OR	95% CI	Adjusted OR	95% CI
**Socioeconomic variables**								
Sex								
Male	1	1	1	1				
Female	1.32	(1.28–1.36)	1.12	(1.07–1.14)	1.27	(1.23–1.31)	1.29	(1.25–1.33)
Age (years)								
19–44	1	1	1	1				
45–64	1.42	(1.38–1.45)	1.00	(0.97–1.02)	1.21	(1.18–1.24)	0.99	(0.97–1.02)
65–74	6.73	(6.49–6.98)	6.57	(6.31–6.83)	7.54	(7.26–7.82)	6.23	(6.00–6.48)
≥75	7.93	(7.57–8.30)	7.22	(6.87–7.58)	9.16	(8.75–9.60)	8.12	(7.75–8.50)
Income ^†^								
≥2000 per month	1	1	1	1				
<2000 per month	1.14	(1.11–1.16)	1.02	(1.00–1.05)	1.07	(1.05–1.10)	1.14	(1.11–1.16)
Occupation								
Professional	1	1	1	1				
Service/physical	1.08	(1.05–1.11)	1.00	(0.97–1.03)	1.05	(1.02–1.08)	1.07	(1.04–1.10)
Others ^‡^	1.25	(1.22–1.29)	1.24	(1.20–1.27)	1.11	(1.08–1.14)	1.24	(1.20–1.27)
Education level								
At least high school	1	1	1	1				
Less than high school	1.23	(1.20–1.26)	1.02	(1.00–1.05)	1.27	(1.24–1.30)	1.31	(1.28–1.34)
Residency								
Metropolitan	1		1		1		1	
Urban	1.07	(1.04–1.10)	1.07	(1.04–1.10)	1.04	(1.01–1.07)	1.04	(1.02–1.07)
Rural	1.35	(1.32–1.38)	1.34	(1.31–1.38)	1.18	(1.15–1.21)	1.21	(1.18–1.24)
Chronic illness ^§^								
None	1	1	1	1				
One	1.29	(1.26–1.33)	1.39	(1.34–1.43)	1.34	(1.30–1.38)	1.38	(1.34–1.42)
Two or more	1.59	(1.49–1.70)	1.62	(1.51–1.73)	1.59	(1.49–1.70)	1.80	(1.68–1.92)
**Health behaviors**								
Health check–up ^††^								
No	1		1		-		1	
Yes	2.48	(2.43–2.54)	2.15	(2.10–2.20)	-		2.28	(2.23–2.33)
Smoking								
Never smoker	1		1		1		1	
Former smoker	1.04	(1.01–1.08)	0.97	(0.93–1.01)	1.03	(1.00–1.07)	1.08	(1.05–1.12)
Current smoker	0.79	(0.77–0.82)	0.74	(0.72–0.77)	0.75	(0.72–0.77)	0.78	(0.75–0.80)
Drinking alcohol								
No	1		1		1		1	
Yes	0.86	(0.85–0.88)	0.87	(0.85–0.89)	0.88	(0.86–0.90)	0.85	(0.83–0.87)
Regular walking								
<30 min/5days/week	1		1		1		1	
≥30 min/5days/week	1.12	(1.10–1.15)	1.14	(1.12–1.16)	1.14	(1.11–1.16)	1.12	(1.10–1.14)

Notes: ^†^ Units: Korean Chun-Won (₩1,000); ^‡^ Others include soldiers, college students, and housewives; ^§^ Patients were diagnosed with one or more of the following diseases by a doctor and were receiving a medication for the disease: diabetes, stroke, heart disease (angina or myocardial infarction), hepatitis, or tuberculosis; ^††^ Variable was excluded from the questionnaire in the 2010–2011 season.

## 4. Discussion

We summarized IVC rates in four consecutive flu seasons in Korea. The large sample of KCHS data revealed a steady increase in self-reported influenza vaccine receipt. The highest increase in vaccination rate was observed between the 2008–2009 and 2009–2010 seasons. This increased vaccine uptake might be explained by the fear of pandemic influenza. Also, in the 2009–2010 season, two vaccines (seasonal and pandemic) were available, which can also explain the increase in IVC rates in the 2009–2010 season.

Overall, IVC increased with age, with the elderly reporting the highest rates of vaccination. People aged 65 years or older had markedly higher vaccination rates than individuals in other countries [29,30,31,32,33,34,35,36], and this group exceeded the WHO vaccination target of 75% [1]. Many other countries have coverage gaps that vary from 4% to 70% between young adults and the elderly [37,38,39,40] and our study revealed great disparities in IVC between these age groups in Korea. Vaccination rates in young adults were 20% to 30%, which is similar to rates in the United States [38]. IVC coverage in people aged 45–64 years was 35.2%, but the rates decreased during the pandemic and then slowly recovered, as shown in Table 1. Coverage gaps between subgroups according to socioeconomic variables differed from other countries. In this study, vulnerable groups such as people of older age, people with a lower education level, people with lower income, people with physical jobs, people residing in rural areas, and females, showed higher rates of IVC. These results are similar to those previously reported from studies in Korea and China [24,29,30]. The highest increase in vaccine coverage was observed in people with lower income. Usually, education levels are low in the elderly, which led to employment in physical jobs with low income, such as those in agriculture and fisheries. However, each of these factors is defined as a vulnerable group in Korea and 253 regional healthcare centers provide free vaccines to vulnerable groups each year. According to studies from other countries, higher education level, higher income, and professional jobs are associated with higher rates of vaccine coverage [31,32,33].

Smoking and drinking alcohol were inversely associated with vaccine receipt, and receiving health check-ups and regular walking were positively associated with vaccination. These results indicate that engaging in health behaviors is a predictor of influenza vaccination; this finding has been previously reported by other studies [30,31,41,42].

When IVC was evaluated in occupation subgroups, higher rates of vaccination were observed in ‘others (soldiers, students, and housewives)’ than in professional and service/physical workers. This may be because the soldiers in the ‘others’ group are obligated to receive the vaccine soon after enlisting in the army. Having a chronic health condition was also positively associated with vaccination, as other studies have reported [24,25,31,34]. Younger people, people with higher income, people with a higher education level, people who do not receive health check-ups, current smokers, and regular alcohol consumers showed a relatively low IVC of approximately 30%. Coverage gaps between subgroups did not narrow and were similar from the 2008–2009 season through the 2011–2012 season. During the influenza pandemic in 2009, many countries offered free vaccination to high-risk groups with campaigns designed to build herd immunity and promote public awareness of the infection. The Korean government prepared vaccines to cover 39% of the total population and offered free vaccination to high-risk groups first [22]. The IVC was lower than expected during the pandemic, but, according to our study, overall IVC rates increased in the year and two years following the pandemic season. This trend is different from other studies that reported that vaccination rates decreased or remained unchanged after the pandemic in China [22,30,37], the United States [38], and Europe [33,39,40,43,44]. One of the reasons that Korea showed comparatively higher IVC might be the NIP, which fully funds the influenza vaccine and administration fee for vulnerable groups (Table 1). The NIP covers more beneficiaries of free vaccination than other countries [22,45]. Additionally, domestically manufactured influenza vaccines might have contributed to high IVC in Korea, since it was easier to distribute a sufficient number of vaccines [46]. The WHO has emphasized ‘sufficient influenza vaccine provision’ as a major factor in increasing IVC [5].

To augment overall IVC, policies targeting young adults will offer the most benefit. In Korea, children and young adults are more commonly infected with influenza and the resulting socioeconomic burden from infection is considerable [47,48,49]. To implement effective interventions that promote public awareness of influenza and reduce the disparities of IVC among subgroups, strategies that focus on these subpopulations must be combined with the current immunization program [50,51].

For this study, we used a large sample of data that comprised approximately 230,000 survey respondents each year. This data is sufficient to provide information on general vaccination coverage rates and trends, but several limitations exist. First, children are a high-risk group for influenza, but IVC in children could not be evaluated in our study because the KCHS only includes adults aged ≥19 years. Therefore, this study does not represent IVC rates of all populations in Korea. Second, self-reported influenza vaccination might be subject to recall bias. Finally, other chronic conditions such as malignancy, renal diseases, and acquired immune deficiency syndrome, which confer a high risk for influenza complications, were not included in the survey [5]. Thus, data on risk groups in this study do not fully represent all of the risk groups in Korea.

## 5. Conclusions

In summary, our results show a steady increase in IVC among adults in Korea from before the pandemic in 2008–2009 to after the pandemic in 2011–2012. IVC rates in Korea are higher than in other countries. However, disparities between subgroups were substantial in people with higher income, people with a higher education level, people who do not receive a health check-up, current smokers, regular alcohol consumers, and young people. Vaccination promotion targeting these specific subgroups should be included in the NIP to increase vaccine coverage. Also, future research should be conducted to define the reasons that young adults abstain from influenza vaccination.

## References

[1] World Health Organization (WHO) (2005). WHO position paper. Wkly. Epidemiol. Rec..

[2] CDC (2013). Prevention and control of seasonal influenza with vaccines. MMWR.

[3] Simonsen L., Fukuda K. (2000). The impact of influenza epidemics on hospitalizations. J. Infect. Dis..

[4] Terebu P., Uyeki T. (2003). Impact of influenza on young children and the shaping of United Sates influenza vaccine policy. Pediatr. Infect. Dis..

[5] World Health Organization (WHO) (2012). Vaccines against influenza WHO position paper—Novermber 2012. Wkly. Epidemiol. Rec..

[6] Jain S., Kamimoto L. (2009). Hospitalized patients with 2009 H1N1 influenza in the United States, April-June 2009. N. Engl. J. Med..

[7] Mallia P., Johnston S.L. (2007). Influenza infection and COPD. Int. J. COPD.

[8] Neuzil K.M., Reed G.W. (1999). Influenza-associated morbidity and mortality in young and middle-aged women. JAMA.

[9] Osterholm M.T., Kelley N.S. (2012). Efficacy and effectiveness of influenza vaccines: A systematic review and meta-analysis. Lancet Infect. Dis..

[10] Puig-Barbera J., Diez-Domingo J. (2012). Effectiveness of the 2011–2011 seasonal influenza vaccine in preventing confirmed influenza hospitalizations in adults: A case-case comparison, case-control study. Vaccine.

[11] Girard M.P., Tam J.S. (2010). The 2009 A (H1N1) influenza virus pandemic: A review. Vaccine.

[12] Song J.Y., Cheong H.J. (2011). Effectiveness of the pandemic influenza A/H1N1 2009 monovalent vaccine in Korea. Vaccine.

[13] Blank P.R., Szucs T.D. (2009). Increasing influenza vaccination coverage in recommended population groups in Europe. Expert Rev. Vaccines.

[14] Simonsen L., Taylor R.J. (2007). Mortality benefits of influenza vaccination in elderly people: An ongoing controversy. Lancet Infect. Dis..

[15] Nicho K.L., Nording J. (2003). Influenza vaccination and reduction in hospitalizations for cardiac disease and stroke among the elderly. N. Engl. J. Med..

[16] Kausz A., Pahari D. (2004). The value of vaccination in chronic kidney disease. Semin. Dial..

[17] Lau D., Eurich D.T. (2013). Effectiveness of influenza vaccination in working-age adults with diabetes: A population-based cohort study. Thorax.

[18] Kramarz P., Ciancio B. (2009). Sesonal and pandemic influenza vaccines for the elderly and other risk groups: A review of available data. Pol. Arch. Med. Wewn..

[19] Lin H.C., Chiu H.F. (2014). Association of influenza vaccination and reduced risk of stroke hospitalization among the elderly. Int. J. Environ. Res. Public Health.

[20] Korea Centers for Disease Control and Prevention (2013). 2013–2014 Instruction Guidelines for Influenza.

[21] OECD Health at a Glance 2011: OECD Indicators, OECD Publishing, 2011. http://dx.doi.org/10.1787/health_glance-2011-en.

[22] Lee Y.K., Kwon Y. (2012). 2009–2010 novel influenza A (H1N1) vaccination coverage in the Republic of Korea. Am. J. Infect. Control..

[23] Heo J.Y., Chang S.H. (2013). Risk perception, preventive behaviors, and vaccination coverage in the Korean population during the 2009–2010 pandemic influenza A (H1N1); Comparison between high-risk group and non-high-risk group. PLoS One.

[24] Ryu S.Y., Kim S.H. (2011). Influenza vaccination among adults 65 years or older: A 2009–2010 community health survey in the Honam region of Korea. Int. J. Environ. Res. Public Health.

[25] Kee S.Y., Lee J.S. (2007). Influenza vaccine coverage rates and perceptions on vaccination in South Korea. J. Infect..

[26] Kim Y.T., Choi B.Y. (2012). Overview of Korean community health survey. J. Korean Med. Assoc..

[27] Rim H., Kim H. (2011). Validity of self-reported healthcare utilization data in the community health survey in Korea. J. Korean Med. Sci..

[28] Oh D.H., Kim S.A. (2013). Prevalence and correlates of depressive symptoms in Korean adults: Results of a 2009 Korean community health survey. J. Korean Med. Sci..

[29] Kee S.Y., Cheong H.J. (2011). Influenza vaccination coverage rate and factors associated with vaccination in people with chronic disease. Infect. Chemother..

[30] Wu S., Yang P. (2013). Influenza vaccination coverage rates among adults before and after the 2009 influenza pandemic and the reasons for non-vaccination in Beijing, China: A cross-sectional study. BMC Public Health.

[31] Takayama M., Wetmore C.M. (2012). Characteristics associated with the uptake of influenza vaccination among adults in the United States. Prev. Med..

[32] Wada K., Smith D.R. (2013). Influenza vaccination uptake among the working age population of Japan: Results from a national cross-sectional survey. PLoS One.

[33] Vaux S., van Cauteren D. (2011). Influenza vaccination coverage against seasonal and pandemic influenza and their determinants in France: A cross-sectional survey. BMC Public Health.

[34] McIntyre A.F., Gonzalez-Feliciano A.G. Flu Vaccination Coverage, United States, 2011–12 Influenza Season. http://www.cdc.gov/flu/pdf/fluvaxview/vax-coverage-1112estimates.pdf.

[35] Bentele H., Bergsaker M.R. (2014). Vaccination coverage for seasonal influenza among residents and health care workers in Norwegian nursing homes during the 2012/13 season, a cross-sectional study. BMC Public Health.

[36] Kroneman M., van Essen G.A. (2006). Influenza vaccination coverage and reasons to refrain among high-risk persons in four European countries. Vaccine.

[37] Zhou L., Su Q. (2013). Seasonal influenza vaccination coverage rate of target groups in selected cities and provinces in China by season (2009/10 to 2011/12). PLoS One.

[38] CDC (2013). Surveillance of influenza vaccination coverage—United States, 2007–08 through 2011–12 influenza seasons. MMWR.

[39] Mereckiene J., Cotter S. (2014). Seasonal influenza immunization in Europe. Overview of recommendations and vaccination coverage for three seasons: pre-pandemic (2008/09), pandemic (2009.10) and post-pandemic (2010/11). Euro. Sruveill..

[40] Caille-Brillet A.L., Raude J. (2013). Trends in influenza vaccination behaviors—Results from the CoPanFlu cohort, France, 2006 to 2011. Euro. Surveill..

[41] Nelson D.E., Bland S. (2002). State Trend in health risk factors and receipt of clinical preventive service among US adults during the 1990s. JAMA.

[42] CDC (2013). Surveillance for certain health behaviors among states and selected local areas—United States, 2010. MMWR.

[43] Pinto C.S., Nunes B. (2013). Trends in influenza vaccination coverage in Portugal from 1998 to 2010: Effect of major pandemic threats. BMC Public Health.

[44] Castilla J., Martinez-Baz I. (2013). Trends in influenza vaccine coverage among primary healthcare workers in Spain, 2008–2011. Prev. Med..

[45] de Lataillade C., Auvergne S. (2009). 2005 and 2006 seasonal influenza vaccination coverage rates in 10 countries in Africa, Asia Pacific, Europe, Latin America and the Middle East. J. Public Health Policy.

[46] Palache A. (2011). Seasonal influenza vaccine provision in 157 countries (2004–2009) and the potential influence of national public health policies. Vaccine.

[47] Kim W.J. (2009). Novel influenza A/H1N1 pandemic: Current status and prospects. J. Korean Med. Assoc..

[48] Kim H.S., Kim J.H. (2011). Fatal cases of 2009 pandemic influenza A(H1N1) in Korea. J. Korean Med. Sci..

[49] Suh M., Kang D.R. (2013). Socioeconomic burden of influenza in the Republic of Korea, 2007–2010. PLoS One.

[50] Yoo B-.K. (2011). How to improve influenza vaccination rates in the U.S. J. Prev. Med. Public Health.

[51] Stinchfield P.K. (2008). Practice-proven interventions to increase vaccination rates and broaden the immunization season. Amer. J. Med..

